# Reconfigurable Diplexer-Based Filtenna for Tx/Rx Operation in Mobile Satellite Terminals

**DOI:** 10.3390/s20082333

**Published:** 2020-04-20

**Authors:** Luís Rodrigues, Tiago Varum, João N. Matos

**Affiliations:** 1 Universidade de Aveiro, Campus Universitário de Santiago, 3810-193 Aveiro, Portugal; luis.carlos.rodrigues@ua.pt (L.R.); matos@ua.pt (J.N.M.); 2 Instituto de Telecomunicações, Campus Universitário de Santiago, 3810-193 Aveiro, Portugal

**Keywords:** reconfigurable antenna, diplexer, filtenna, microstrip antenna, PIN diode, LEO satellites constellation, IoT

## Abstract

Large constellations of low-orbit satellites have already been launched with the aim of offering complete worldwide coverage of broadband Internet; however, compact, simple, and low-cost mobile terminals are necessary to establish the communication. This paper describes the design of a reconfigurable and compact filtenna with the ability to switch between the satellites’ uplink and downlink frequencies, 20 GHz and 29 GHz, maintaining an excellent performance. Due to its simplicity, efficiency, and Rx/Tx isolation, this antenna is a relevant candidate to be part of mobile terminals and devices, or even sensors, that communicate with satellites.

## 1. Introduction

The development of communications systems has followed the technological advances and the pressure that societies exert on better services, amenities, and being online. More and more mobile terminals and devices are being connected, which increase the challenges of the entire communications network. In addition, the population increasingly consumes more information, multimedia, and digital services offered to them, and it does not give up these privileges.

The new generation of mobile communications (5G) intends to respond to these technological challenges, allowing an increase in the density of connected devices, as a result of a tremendous capacity growth, providing wider bandwidths for higher data transmission rates with much lower latency. As a result of this new communications technology, and its capabilities, the Internet of Things (IoT) concept can be implemented by connecting devices, and devices with people, quickly and effectively through a broad intelligent communications network.

Nowadays, there is a major worldwide commitment by several companies to provide a global coverage of the planet with affordable broadband Internet, even in the poorest or most remote areas, using low-orbit satellite constellations. A significant percentage of people do not have access to the Internet, or do not have access to high-quality Internet, as it is impossible to have infrastructures for the entire world, which are expensive and complex [1].

Compared to geostationary, low-orbit satellites require less power in the communication between the transmitter and the receiver, lowering costs and enabling the use of low-cost terminals on the ground. Besides a greater simplicity in the process of launching and placing satellites in orbit, the latency of the communication is substantially reduced, making them suitable for real-time critical applications.

**Figure 1:**
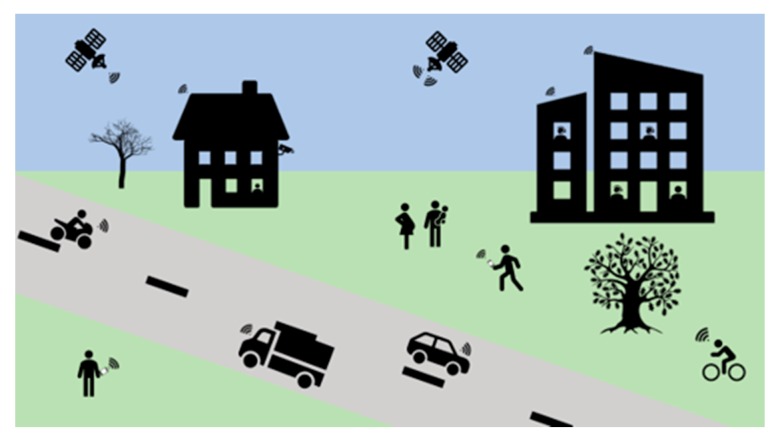
Smart city/Internet of Things (IoT) scenario and satellite communications.

For these reasons, the use of satellites is a crucial point in the technological evolution. They will allow global coverage of broadband internet at controlled costs, and their inclusion or support in the modern communication networks, 5G or subsequent, contributing to the implementation of the concept of the Internet of Things and smart cities, where everyone is online and connected, as illustrated in Figure 1.

To allow the communication with the low-orbit satellite constellations, mobile terminals must adapt their characteristics to operate according to the satellite’s requirements, such as their frequency bands. The communications are made at high frequencies (Ka band), using different bands for the uplink (27.5–30 GHz) and downlink (19.7–21.2 GHz). It is essential that mobile devices maintain their simplicity, compactness, efficiency, and low cost, but with the ability to operate in the two different frequency bands.

Antennas are an essential component of any wireless communication system. Its development needs to be constant to keep up with new technological challenges and to accommodate different properties in terms of communication. In case of mobile devices using satellite communications, their antennas must be able to operate in the different satellite bands (for Tx and Rx). In addition, more compact devices, more powerful and with a wider range of communications are increasingly in demand; however, the space available is growing increasingly smaller. An antenna that is capable of operating in two different bands, replacing the need for two antennas, saving space, is an important benefit for modern systems. These antennas with the ability to adapt one or more of their operating parameters are referred to as reconfigurable antennas.

Reconfigurable antennas can change their properties, such as resonating frequency, gain, radiation pattern, or polarization, according to the communication requirements. A single element can do the same work that would be achieved using multiple elements. To accomplish this adjustment, the physical structure of the antenna can be modified, such as the feeding network or the radiating element, leading to a changing in the current distribution. The reconfigurability can be obtained using some techniques, being popular those using varactors, as tuning elements, or RF-MEMS (Radio Frequency-Micro Electro Mechanical System) and PIN diodes which can act as ON/OFF switches, changing the physical structure of the antenna.

In [2], a microstrip patch is presented with the ability to operate at three different bands: 2.3–2.51 GHz, 3.35–3.75 GHz, and 4.95–5.53 GHz. To achieve the reconfigurability, PIN diodes were inserted in the ground plane, which can connect or disconnect different metal structures that allow the adjustment of the operating frequency. A reconfigurable antenna that is capable of changing its radiation pattern and resonating frequency is shown in [3]; this antenna is composed by a circular slot with a PIN diode in the top side of the substrate, allowing the change of the radiation pattern. In the opposite side of the substrate, there is the feed line with a stub, which can be connected to the main line by PIN diodes. So, controlling the state of the switches, it is possible to define if the stubs are or are not connected to the central line, controlling the matching of the antenna and the resonating frequency. A polarization reconfigurable antenna can be seen in [4], where a circular slot was integrated in a circular microstrip patch; two PIN diodes were inserted between the slot and one of the antennas edges, and if they are connected, it is possible to select the polarization of the antenna. Another technique using RF-MEMS can be seen in [5], where an antenna that can change its radiation pattern is presented, operating at 6.85 GHz.

Analyzing the reported works, it is possible to conclude that the presented antennas cannot be integrated into mobile terminals to communicate with satellites constellations. In general, they operate at low frequencies, the bandwidth is narrow, the range of frequencies is small, they need coaxial cables for power supply, and they use capacitors that do not work properly when the frequency increases. In addition, besides the low frequency, in some examples, the complexity is high.

In [6], an array of some slots in a “T” shape is shown. The slots allow the antenna to operate at two high frequencies: 28 GHz and 38 GHz. PIN diodes were inserted in both arms of each slot in order to achieve the reconfigurability. So, depending on the diodes state (on or off), the antenna can change between the two frequencies. Another reconfigurable antenna is shown in [7]. This one has two ports: one for the reception (23 GHz until 24 GHz) and another for transmission (25 GHz until 27 GHz and 27 GHz until 30 GHz). Two band pass filters with radial stubs were designed to block the frequencies outside from the bands mentioned above. Since for transmission two bands were allowed, a PIN diode was inserted inside the filter to allow the change between both bands.

In [8], the authors present four models of microstrip patch antennas operating at 60 GHz. Even though these antennas were designed on the same substrate, the patches and ground plane are different. Besides that, they have slots using different shapes with variable resistors inside, and changing the value of the resistors, it is possible to modify some of antenna’s characteristics, such as resonance frequency, bandwidth, or gain. An ultra-wide bandwidth antenna that is capable of operating within the range of 3.1 to 10.6 GHz is reported in [9], using a band pass filter consisting of short-circuited shunt stubs and inter digital edge coupled microstrip lines, to improve the selectivity, since the frequencies out of this band can generate interference with others communication systems. In [10], a novel broadband antenna is presented using a band pass hairpin filter that is integrated in the structure, filtering between 2.03 and 2.3 GHz. The microstrip patch acts both as a radiating element and as the last resonator of the band pass filter, allowing the antenna to radiate within the filter frequencies.

In this paper, a small reconfigurable microstrip patch filtenna is presented that can be integrated into mobile devices to communicate with low-orbit satellites. The filtenna can operate in two different bands, 20 GHz and 29 GHz. To change the operating frequency, two different metal structures can be connected via a PIN diode. Two hairpin filters were used to eliminate the undesired frequencies, and they also work as a DC blocker.

This paper is organized in five sections. In Section 1, an introduction is presented, followed by Section 2, where the choice of the filters and the switching element is described. In Section 3, the reconfigurable filtenna design is explained, and in Section 4, the measured and simulated results are shown. Finally, the conclusion of this work is presented in Section 5.

## 2. Structure and Reconfigurability

The proposed antenna is based on microstrip structures, as they allow designing efficient, versatile, compact, and simple elements, with low manufacturing cost [11]. Microstrip structures consist of a dielectric substrate covered on both sides by conductive material; typically, one side is completely covered, functioning as a ground plane, while the other side is only partially filled, delimiting the radiating surface.

To achieve the reconfigurability, a technique from the three mentioned before was chosen. RF-MEMS were discarded, since they need a high bias voltage to commute between the ON/OFF states and present a high overall cost [5,12]. As varactors are highly nonlinear, PIN diodes were used, since they require low bias voltages, are easy to find on the market at low cost, and the switching speed is fast [13].

To suppress the unwanted frequencies, both the transmitted and received signal were filtered. As the frequencies are high, the undesired bands can influence the behavior of the antenna, affecting its performance. The nonlinear behavior of the PIN diode was taken into account, as it can reproduce harmonics that reduce energy from the system. Another important point is the fact that DC voltage will be applied to the diodes. Therefore, to separate the DC signal from the RF signal, filters that do not have directly connected copper pieces are a good solution.

Some filters were considered such as an interdigital filter, combline filter, pseudo combline filter, end-coupled, edge-coupled, or a hairpin filter. These filters are designed with λ/2 or λ/4 lines, differing just in their layout and some features. The hairpin structure was selected since it presents wider bandwidth [14]. Considering that the radiating physical structure comprises an antenna and includes a filter, it is common for this arrangement to be called a filtenna.

## 3. Antenna Design

The design of the filtenna started with the construction of the radiating element, the reconfigurable antenna. A microstrip main patch was designed to the higher frequency, followed by the design of a secondary metal structure that allows the antenna to operate at the satellite receiver (lower) frequency band, when connected to the main patch. A rectangular microstrip patch structure was used as it is simple and the feeding techniques are widely known and easy to implement.

To adapt the same antenna to two different frequencies, the inset feed technique was selected. This method consists of creating a reentrance in the main patch until the input impedance equals the characteristic impedance of the feed line [11].

**Figure 2:**
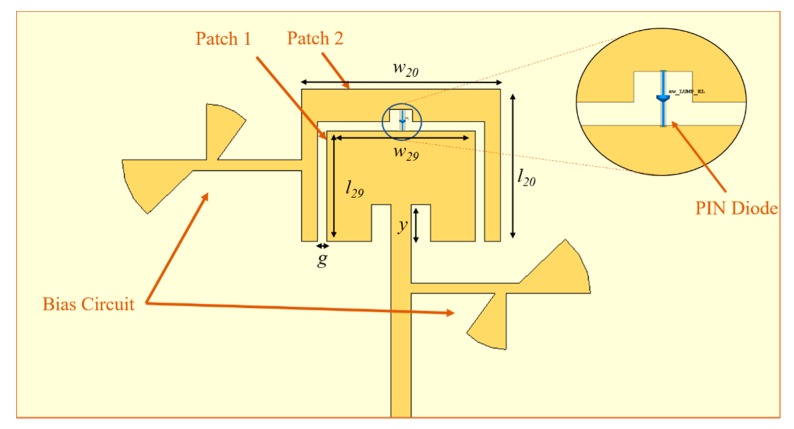
Dual frequency microstrip antenna structure (main patch and outer structure) using a PIN diode and two bias circuits.

The main rectangular patch that allows the antenna to operate at 29 GHz was designed first with the dimensions of 2.41 × 3.25 mm^2^. The matching was made to 90 Ω, since this impedance allowed having a feed line with an adequate (thin) width, weighing its dimensions, the feasibility, and the losses of the structure. A second rectangular metal structure was designed around the main patch and, when both are connected via a PIN diode, the antenna resonates at 20 GHz. The structure of the antenna can be observed in the Figure 2, and its main dimensions are presented in Table 1.

The dielectric substrate used was the RO4350B, with a dielectric constant ε_r_ of 3.48, thickness of 0.762 mm, and loss tangent of 0.0037 (@10 GHz).

The frequency reconfigurability is achieved by connecting or disconnecting the main patch (Patch 1) from the outer structure (Patch 2) through a switch, which can be in an ON or OFF state. So, if the diode is reversed-biased (OFF), the patch is disconnected, and the antenna operates at 29 GHz. Otherwise, if the diode is forward-biased (ON), the patches are connected, working as a single patch, and the structure is resonating at 20 GHz.

**Figure 3:**
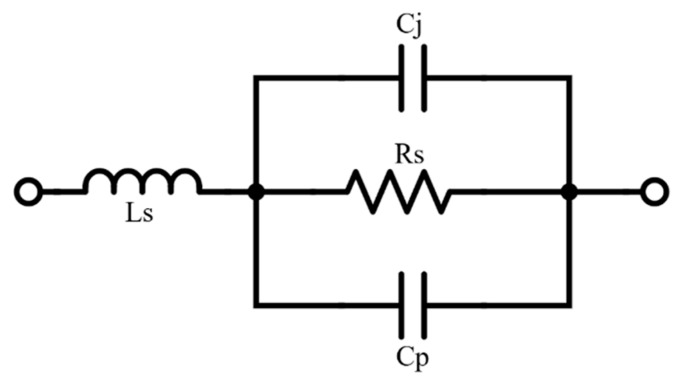
PIN diode equivalent model.

The switching element was the MA4PBL027 [15], which is a MACOM silicon beam PIN diode. This element has low parasitic elements: series resistance, capacitance, and inductance. The switching speed is extremely fast and has low insertion loss and high isolation, making it perfect to be used in microwave and millimeter wave switch designs. Its equivalent model is shown in Figure 3, and it was used in the electromagnetic simulator.

The intrinsic parasite values of this model correspond to a maximum series resistance of 4.0 Ω, a series inductance of 0.15 nH, a junction capacitance of 0.04 pF, and a parasitic capacitance of 8.0 fF.

**Figure 4:**
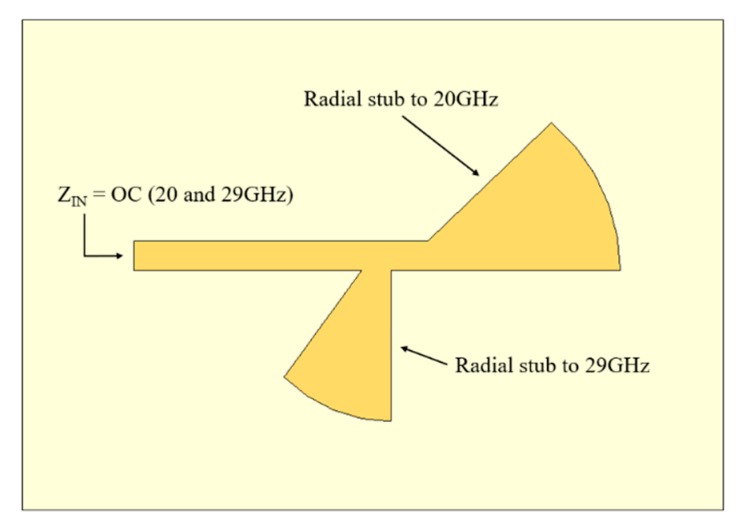
Designed structure of the dual frequency bias circuit to the PIN diodes.

To turn the diode forward-biased or reverse-biased, it is necessary to apply a DC voltage to its terminals. A bias circuit was designed to supply this voltage to the switch diode, which was composed of radial stubs and λ/4 transformers. It is crucial that this circuit does not affect the performance of the antenna, and so, the RF signal should not cross it. To accomplish that, the input impedance of the bias circuit should be extremely high, that is, theoretically infinite, corresponding to an open circuit (OC) for both frequencies (20 GHz and 29 GHz). Therefore, the circuit acts as an RF blocker, preventing the RF signal from reaching the DC source. Figure 4 shows the structure of the designed dual-frequency bias circuit.

To place the bias circuit in the structure of the antenna, a detailed parametric study was made in order to identify the most appropriate location so that it does not affect the performance of the antenna. Regarding the bias circuit attached to the patch, it was confirmed by simulation that the best position was the center of the patch length, as it is the location where the patch has the lowest impedance (theoretically zero), and therefore, the appropriate location to place in parallel an extremely high impedance, minimizing any impact. As for the bias circuit added to the patch’s feed line, several simulations were carried out varying its location, evaluating the place where this copper structure would have the least effect on the overall characteristics of the antenna.

**Figure 5:**
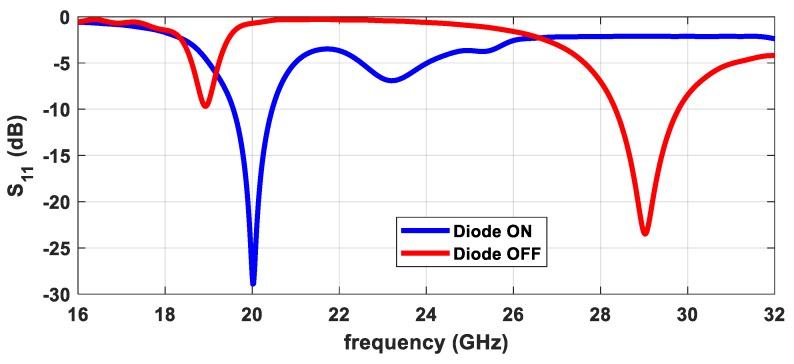
Simulated S_11_ of the individual patch antenna for both states of the PIN diode.

Once the diode is placed on the antenna, Figure 5 shows the simulated response of the projected antenna (presented in Figure 2) for the two states of the diode, ON and OFF, for which the antenna operates in the 20 and 29 GHz band, respectively.

It is possible to verify that the antenna is appropriately matched for both states, with an S_11_ lower than −24 dB, and for a theoretical 90 Ω impedance characteristic of the line, the simulated value for the input impedance is 84 Ω@20 GHz and 103 Ω@29 GHz, which is an excellent compromise for both frequencies.

**Figure 6:**
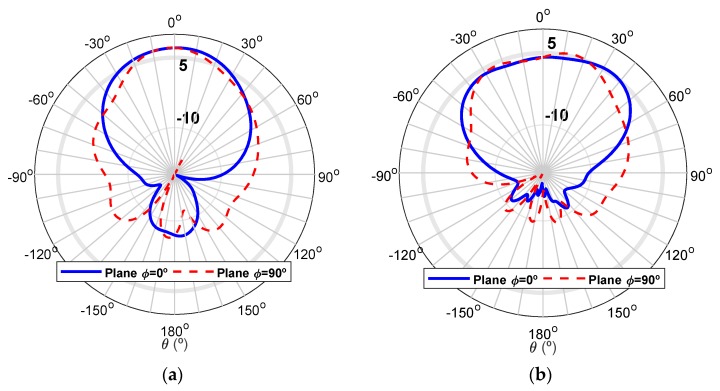
Simulated radiation pattern of the individual patch antenna in the two main radiation planes, for both states of the PIN diode: (**a**) ON mode (20 GHz) and (**b**) OFF mode (29 GHz).

Figure 6 shows the simulated radiation pattern of the microstrip patch, in the two main planes, for the two states of the diode: (a) ON—20 GHz (b) OFF—29 GHz. It is possible to verify that the element maintains an almost semi-spherical radiation, which is typical of these antennas, with a maximum gain of 7.12 dBi at 20GHz and 5.45 dBi at 29GHz.

The microstrip hairpin filter can be used at microwave and millimeter-wave frequencies. Its name comes from its “U” shape and it is composed by λ/2 folded parallel coupled lines. With this topology, it is possible to obtain greater bandwidths with a small structure, since compared with others filter topologies, the hairpin filter is more compact [14]. To design the filter, some issues must be considered, such as its order, since it is a compromise between the size and performance.

**Figure 7:**
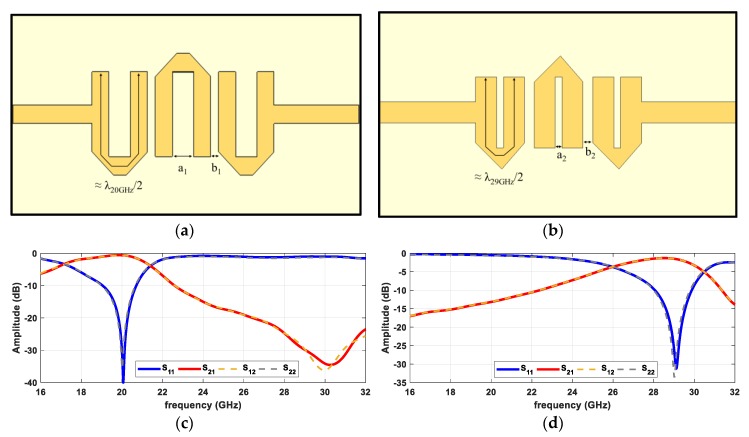
Structure of hairpin filters and its S-Parameters simulation: (**a**) filter in 20 GHz band; (**b**) filter in 29 GHz band; (**c**) 20 GHz hairpin filter response; and (**d**) 29 GHz hairpin filter response.

In this filtenna, two third-order hairpin filters were designed, one for each frequency (20 GHz and 29 GHz) with Z_0_ = 90 Ω transmission lines, as it is the characteristic impedance of the antenna’s feed line. Each U section comprising the filter is a λ/2 microstrip line folded with a separation between ends of a_n_ and spaced from the next units of b_n_. As they are third-order filters, it is possible to observe three ‘U’ sections. The structures of the two designed hairpin filters are shown in Figure 7a,b.

The two microstrip filters were designed and optimized in the electromagnetic simulator to find the ideal dimensions of the gaps (a_n_ and b_n_) ensuring proper operation. The final dimensions of the filters designed using 90 Ω characteristic impedance microstrip lines are a_1_, b_1_, a_2_, and b_2_ of 0.54 mm, 0.2 mm, 0.14 mm, and 0.2 mm, respectively. These values allowed creating filters with good performance, maintaining a good reflection coefficient of the overall structure in both terminals, and a good transmission in the desired band, which are properties that can be observed in Figure 7c,d.

**Figure 8:**
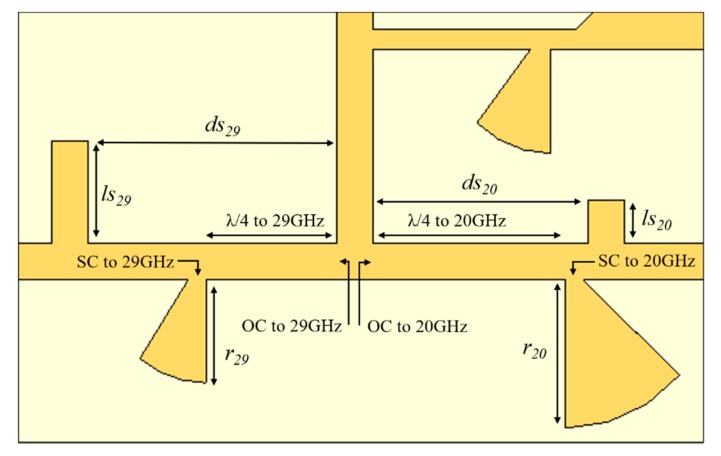
Microstrip feeding structure of the dual-frequency antenna using two stop-band filters at each frequency (open circuit, or OC: radial strub + λ/4 line).

To associate two signals of different frequencies to the same antenna, a method to isolate them had to be found. Two structures working as a two stop-band filters were designed and integrated in the signal paths, as shown in Figure 8.

The main objective of using these band-stop filters was to isolate the two bands, that is, preventing that the downlink signal (20 GHz), received by the antenna not to reach port 2, going directly to port 1. Similarly, in transmission mode (29 GHz), it was avoiding the signal from port 2 to reach port 1, going directly to the antenna.

To build such a blocking structure, to 29 GHz, a stop-band filter was designed that ensures that when this signal reaches the intersection with the antenna’s feed line, it will see an extremely high impedance in the port 1 direction. This way, most of the signal will follow the antenna. For a better understanding, the structure is shown in Figure 8. To design this stop-band filter, a radial stub that forces a short circuit (SC) to 29 GHz was used followed by a λ/4 length line at the same frequency, allowing an open circuit (OC) at the end of this line. So, the signal will observe high impedance (theoretically infinite), and if the diode is reversed-biased, the antenna will radiate it. To improve the antenna matching, an open stub between the 20 GHz radial stub and the 29 GHz hairpin filter was placed, reducing the reflection of the 29 GHz signal.

Regarding the downlink signal, the same analysis can be done; when the signal reaches the intersection with the antenna’s feed line, it will see an OC to 20 GHz toward port 2, being received through port 1. A radial stub at 20 GHz is placed at λ/4 distance of the line’s intersection, creating high impedance. In addition, an open stub was inserted between the 29 GHz radial stub and the 20 GHz hairpin filter to improve the antenna matching. The stop-band filter structure was simulated and optimized, and its final dimensions are reported in Table 2.

Using the technique described above, it was possible to develop a reconfigurable printed antenna that is capable of operating in two different frequency bands, at 20 GHz and 29 GHz, connected to two independent transmission channels, and therefore two ports, through the activation of a PIN diode. This configuration also allows guaranteeing the isolation between bands through the inclusion of two filters in the path of the antenna feeding, making it a filtenna.

**Figure 9:**
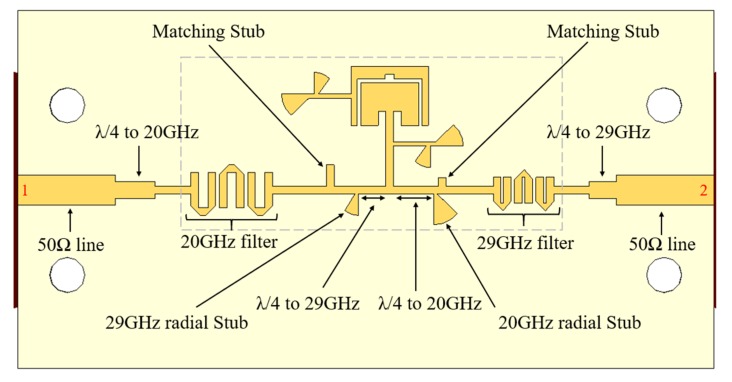
Complete structure of the designed reconfigurable filtenna (dual-band antenna and feeding arrangement with filters).

The designed filtenna is presented in Figure 9. Note that the structure that is going to be integrated into mobile devices is the one limited with the gray dashed line with the dimensions of 21.9 × 9.8 mm^2^.

As it is possible to see in Figure 9, the filtenna is composed of a main patch surrounded by an outer metal structure, with the ability to operate in the satellite’s downlink and uplink bands. Two λ/4 length transformers for both frequencies (with characteristic impedance of 67 Ω) were used to convert the 90 Ω to the standard 50 Ω lines. The red numbers correspond to the numbers that identifying the two ports.

## 4. Results and Discussion

**Figure 10:**
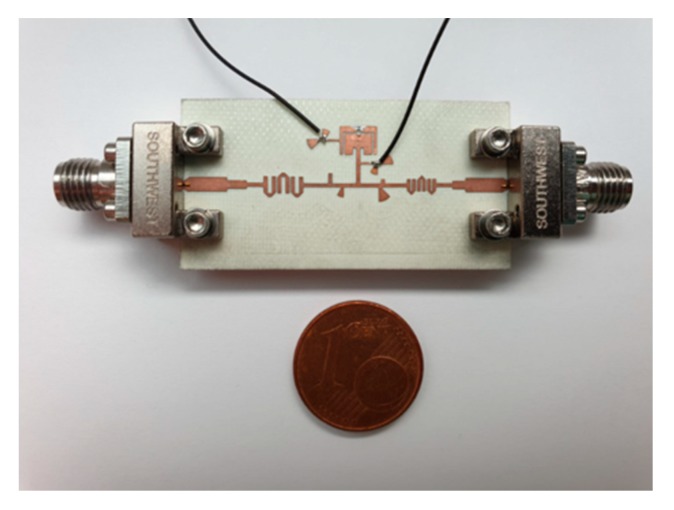
Photography of the fabricated filtenna prototype.

The projected reconfigurable filtenna was manufactured, and the prototype is shown in Figure 10. In this section, the antenna is characterized in terms of its main properties, for both operating bands (20 GHz and 29 GHz), through simulation and measurements results. Port 1 was used for the downlink band and port 2 was used for the uplink band.

**Figure 11:**
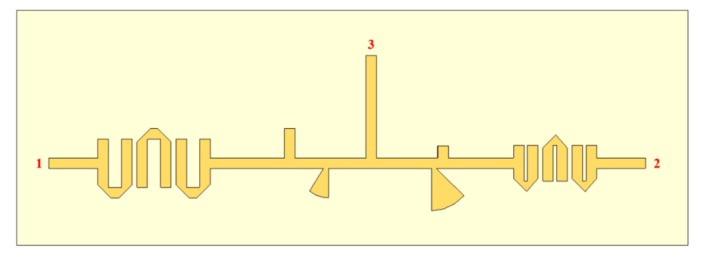
Structure of antenna feeding using filters.

**Figure 12:**
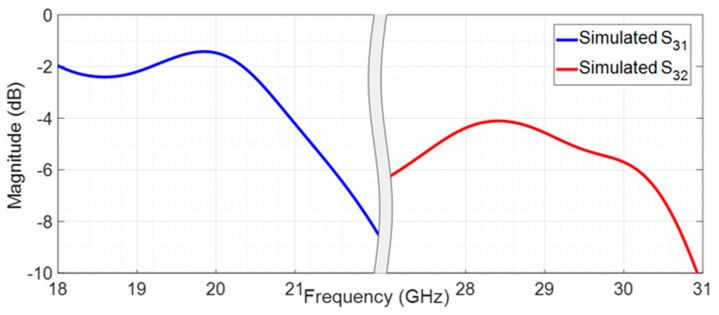
Simulated losses of the filtenna feeding structure.

The filter structures combined in the antenna feeding network, as presented in Figure 11, were simulated to analyze the losses of the structure, and the results are shown in Figure 12.

Figure 12 shows the attenuation of the signals (in both bands 20 GHz and 29 GHz) in the feeding structure (filters and microstrip lines). It is possible to observe that the uplink signal suffers an attenuation of 4.6 dB until it reaches the antenna and the downlink signal suffers an attenuation of 1.5 dB until it reaches port 1.

**Figure 13:**
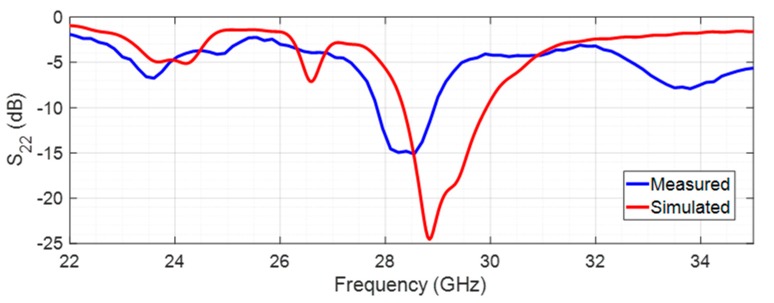
Simulated and measured reflection coefficient of the filtenna in transmission mode.

Figure 13 presents the comparison between the simulated and measured reflection coefficient in port 2, in the transmission mode (operating at 29 GHz).

Analyzing Figure 13, it is possible to observe a similar behavior of both simulated and measured curves, despite a slight deviation in the central resonance frequency. Regarding the simulation results, it can be concluded that the filtenna is properly adapted with a reflection coefficient of −21.3 dB at 29 GHz. The simulated bandwidth is 1.58 GHz (28.35–29.93 GHz). In terms of the measured results, the antenna at 28.55 GHz has an S_22_ of −15 dB with a bandwidth of 1.1 GHz (27.85–28.95 GHz). The main discrepancies could be explained by possible inaccuracies in the manufacturing process, due to the small dimensions of the structure.

**Figure 14:**
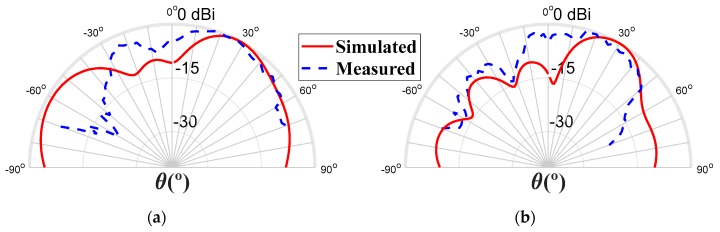
Radiation pattern to 29 GHz, (**a**) Plane φ = 90° and (**b**) Plane φ = 0°.

In Figure 14, the simulated and measured normalized radiation patterns are shown for the two main radiation planes, at 29 GHz. It is possible to see an adequate correspondence between both curves. The simulated gain was 7.41 dBi, and the measured gain was 4.61 dBi. Some deviations can be explained by the impact of connectors and the DC wires in the radiation.

**Figure 15:**
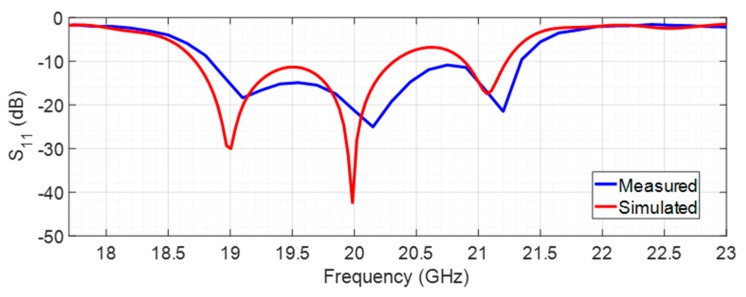
Simulated and measured reflection coefficient of the filtenna to 20 GHz.

When the PIN diode is forward-biased, the filtenna changes the operation frequency band to 20 GHz for the receiving mode. In such case, the comparison between the simulated and measured S_11_ is shown is Figure 15.

According to Figure 15, the simulated S_11_ is −42.4 dB at 20 GHz, and its measured value is −21.3 dB (also at 20 GHz) with a bandwidth of 2.55 GHz (18.8–21.35 GHz), assuming the commonly used criteria of S_11_ < −10 dB.

**Figure 16:**
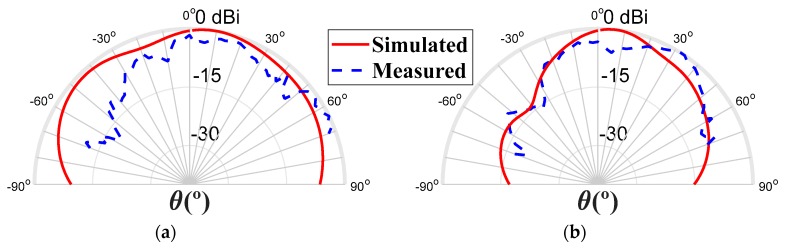
Radiation pattern to 20 GHz, (**a**) plane φ = 90°, and (**b**) plane φ = 0°.

Figure 16 shows the comparison between the simulated and measured radiation patterns for both planes, at 20 GHz. It is possible to notice an identical performance in both curves. The simulated gain was 5.62 dBi, and the maximum measured gain was 4.01 dBi.

**Figure 17:**
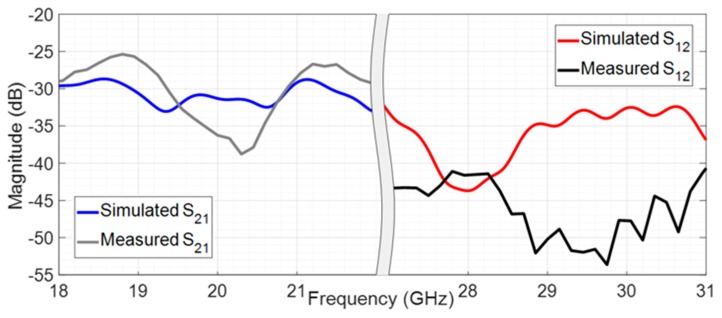
Simulated isolation between two ports.

An important measure in a two port system, such as this designed structure, is the isolation between the two ports, as provided in Figure 17, which reflects the level of effect that a signal on one port has on the opposite port.

When the PIN diode is ON and the antenna is in receiving mode (at 20 GHz), it is possible to conclude that the simulated and measured values are similar, and the isolation is higher than 30 dB at 20 GHz, and it is always above 25 dB in the evaluated band. These results were expected since the downlink signal should be sent all the way to port 1. In transmitting mode, with the PIN diode OFF, the signal injected in port 2 is almost all transmitted and just a negligible part is observed in port 1, since both simulated and measured results reveal an attenuation always higher than 33 dB in the entire band, being the measured S_12_ at 29 GHz of −50.2 dB.

**Figure 18:**
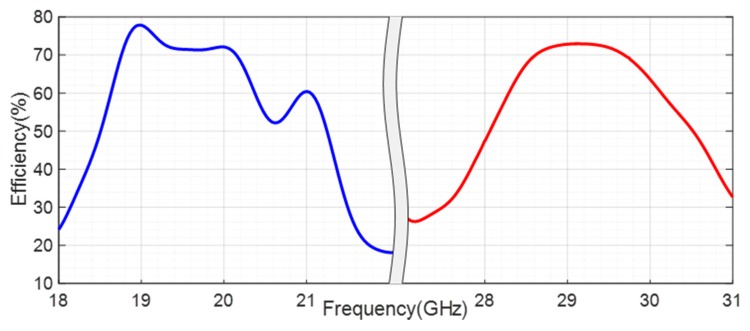
Simulated total efficiency of the filtenna.

Figure 18 shows the simulated efficiency of the designed antenna, which reaches the value of 72% at 20 GHz and is of 73% at 29 GHz for the transmission.

## 5. Conclusions

In this paper, a reconfigurable filtenna is presented operating in the two Ka bands of satellite communications. This antenna is suitable to low-cost mobile terminals that communicate with low-orbit constellations. This simple antenna can switch between two distinct frequency bands, in an efficient way, which can save space. Two filters were integrated into the structure to eliminate the undesired frequencies and allow an Tx/Rx isolation higher than 35 dB at the central frequencies, improving the performance. The antenna is highly compact, with an area of 21.9 × 9.8 mm^2^, and quite efficient with values above 70%. The structure is inexpensive, easy to manufacture, and the bias voltage used to change the PIN diode state is low, which are important benefits for its use in mobile terminals and IoT sensors.

## Figures and Tables

**Table 1:** Main dimensions of the designed microstrip antenna structure (in mm).

*l*_20_	*w*_20_	*l*_29_	*w*_29_	*y*	*s*
3.32	4.33	2.41	3.25	0.79	0.2

**Table 2:** Main dimensions of the matching and stop-band filter structure (in mm).

*ls*_20_	*ds*_20_	*ls*_29_	*ds*_29_	*r*_20_	*r*_29_
0.50	2.51	1.20	2.91	1.73	1.20
